
JOURNAL INFORMATION
==============================
NLM Title Abbreviation: ChemistryOpen
Journal ID: ChemistryOpen
NLM Journal ID: 101594811
Journal ID: open

ChemistryOpen

EISSN: 2191-1363
Publisher: Wiley

ARTICLE INFORMATION
==============================
PMCID: PMC3594583
PMID: 24551520
DOI: 10.1002/open.201390003
Article version: 1
Subjects: Graphical Abstract

Graphical Abstract: ChemistryOpen 1/2013

Print publication date: 2013 Feb
Electronic publication date: 2013 Feb 27
Volume: 2
Issue: 1
First page: 7
Last page: 8
Copyright: Copyright © 2013 WILEY-VCH Verlag GmbH & Co. KGaA, Weinheim
Copyright year: 2013
License: Re-use of this article is permitted in accordance with the Creative Commons Deed, Attribution 2.5, which does not permit commercial exploitation.
License URL: https://creativecommons.org/licenses/by/2.5/

==============================
### COVER PICTURE

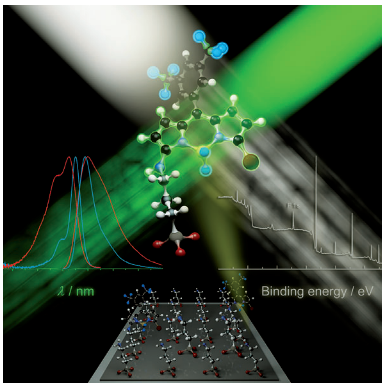

Retrieving quantitative molecular information from macroscopic areas in a reliable manner at micrometric resolution is a challenging task, yet essential for a better understanding and control of the assembly of (bio)chemical entities on a functional surface. In their Full Paper, Knut Rurack and co-workers introduce fluorine-containing moieties to boron-dipyrromethene (BODIPY) dyes, thus making them versatile probes for dual-method surface analysis (p. 25 ff.).
**The cover picture shows** a bright fluorescent dye (green halo) containing a high number of fluorine atoms (blue) for the assessment of reactive amino groups on a glass surface by two complementary surface analytical techniques, non-destructive fluorescence scanning and quantitatively traceable X-ray photoelectron spectroscopy (XPS), both applied to the same individual samples.

### COVER PROFILE

*"The present work is a good example of tailor-made functional dyes with specific properties allowing for (at least) dual-method detection."*
This and more about the story behind the cover research can be found on p. 25.

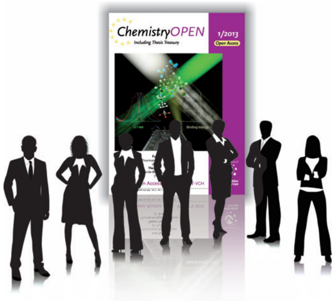

*M. Hecht, T. Fischer, P. Dietrich, W. Kraus, A. B. Descalzo, W. E. S. Unger, K. Rurack**

***2***

**Fluorinated Boron-Dipyrromethene (BODIPY) Dyes: Bright and Versatile Probes for Surface Analysis**

### EDITORIAL

How, as a Chemist, Can I Find Funding for Open-Access Publishing?
This information is crucial to you, as author of an open-access paper.
Read more by the Editors on p. 3 ff.

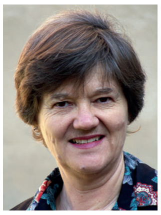

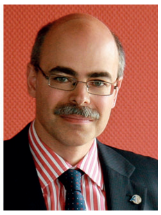

*K. J. Hindson, H. Ross**

***3–5***

**Show Me the Money!**

### NEWS

Spotlights on our sister journals ***10–12***

### THESIS TREASURY

**Fluorine is unique!** A synthesis method for polynuclear systems with bridging fluorido ligands has been developed. The method is based on anchoring and protection of the fluorido ligand by initial coordination to a robust metal center (e.g., $Cr^{III}$) and subsequent use of the robust fluoride complex for construction of more complicated polynuclear structures.

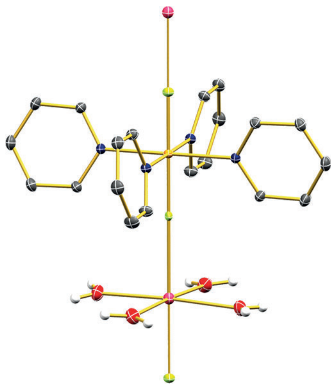

*T. Birk**

***13–16***

**Fluoride as Ligand: Chemistry of Some New Terminal and Bridged Systems**

© 2013 Wiley-VCH Verlag GmbH & Co. KGaA, Weinheim
WILEY ONLINE LIBRARY

### COMMUNICATIONS

*J. Jeromenok, W. Böhlmann, C. Jäger, J. Weber**

***17–20***

**Carbon Dioxide Adsorption in Betulin-Based Micro- and Macroporous Polyurethanes**

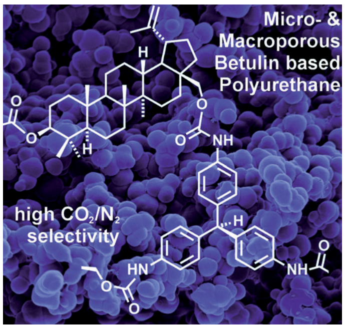

**Separation of $CO_2$.** Microporous polyurethane networks were prepared based on a renewable resource. Betulin, extracted from birch bark, is used as a structure-directing diol monomer in $A_3$-$B_2$ monomers. The resulting microporous networks show very promising $CO_2/N_2$ selectivities. The state of adsorbed $CO_2$ is analyzed by in situ NMR spectroscopy, and pure physisorption is proven. The preparation of monolithic materials is demonstrated as well.

*A. Mazare, N. Liu, K. Lee, M. S. Killian, P. Schmuki**

***21–24***

**Embedded Palladium Activation as a Facile Method for $TiO_2$-Nanotube Nanoparticle Decoration: $Cu_2O$-Induced Visible-Light Photoactivity**

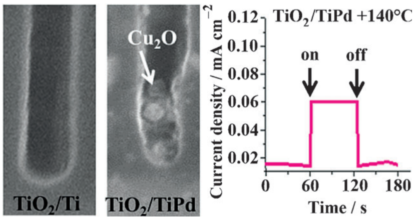

**Highly decorated!** A direct and efficient way to decorate $TiO_2$ nanotubes with copper, $Cu_2O$ and CuO particles is shown. Active palladium species embedded in the $TiO_2$ nanotube walls allow specific initiation sites to be triggered for electroless copper deposition. These $Cu_2O$-decorated $TiO_2$ nanotubes could be used for water splitting.

### FULL PAPERS

*M. Hecht, T. Fischer, P. Dietrich, W. Kraus, A. B. Descalzo, W. E. S. Unger, K. Rurack**

***25–38***

**Fluorinated Boron-Dipyrromethene (BODIPY) Dyes: Bright and Versatile Probes for Surface Analysis**

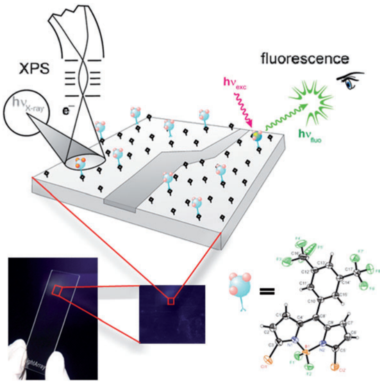

**Give'em fluorine!** Bright and highly photostable boron-dipyrromethene (BODIPY) dyes were obtained by the introduction of up to three pentafluoro- or 3,5-bis(trifluoromethyl)phenyl moieties to the BODIPY core, which present a first generation of potent dual X-ray photoelectron spectroscopy (XPS)/fluorescence labels for the quantification of surface functional groups.

* Author to whom correspondence should be addressed.

Supporting information on the WWW (see article for access details).

This is an open-access article, published under the terms and conditions of the Creative Commons Attribution Non Commercial License.

A video clip is available as Supporting Information on the WWW (see article for access details).

VIP Contributions labeled with this symbol have been judged as "Very Important Papers" by the referees.

### SERVICE

**All the Tables of Contents may be found on the WWW under: http://www.chemistryopen.org**

© 2013 Wiley-VCH Verlag GmbH & Co. KGaA, Weinheim
